# Series 3: From Infection to Disease: A Global Scoping Review of Medical and Behavioural Determinants of Progression from TB Infection to TB Disease

**DOI:** 10.3390/tropicalmed11040094

**Published:** 2026-04-02

**Authors:** Sonia Menon, Anthony D. Harries, Riitta A. Dlodlo, Gisèle Badoum, Mohammed F. Dogo, Olivia B. Mbitikon, Pranay Sinha, Yan Lin, Jyoti Jaju, Aung Naing Soe, Anisha Singh, Bharati Kalottee, Kobto G. Koura

**Affiliations:** 1 International Union Against Tuberculosis and Lung Disease, 75001 Paris, France; soniasimonemenon@gmail.com (S.M.); adharries@theunion.org (A.D.H.); rdlodlo@theunion.org (R.A.D.); gisele.badoum.consultant@theunion.org (G.B.); fall.dogo.consultant@theunion.org (M.F.D.); olivia.mbitikon.consultant@theunion.org (O.B.M.); psinha@bu.edu (P.S.); ylin.consultant@theunion.org (Y.L.); jyoti.jaju@theunion.org (J.J.); ansoe08@gmail.com (A.N.S.); anisha.singh@theunion.org (A.S.); bharati.kalottee@theunion.org (B.K.); 2 Epitech Research, 1160 Auderghem, Belgium; 3 Department of Clinical Research, Faculty of Infectious and Tropical Diseases, London School of Hygiene and Tropical Medicine, London WC1E 7HT, UK; 4 Unité de Formation et de Recherche en Sciences de la Santé (UFR/SDS), Université Joseph KI-ZERBO, Ouagadougou P.O. Box 7021, Burkina Faso; 5 Service de Pneumologie, Centre Hospitalier et Universitaire de Yalgado Ouédraogo (CNHU-YO), Ouagadougou P.O. Box 7022, Burkina Faso; 6 Section of Infectious Diseases, Boston University Chobanian and Avedisian School of Medicine, Boston, MA 02118, USA; 7 Boston Medical Center, Boston, MA 02118, USA; 8 Unité Mixte de Recherche MERIT (UMR261), Université Paris Cité, Institut de Recherche pour le Développement (IRD), 75006 Paris, France

**Keywords:** tuberculosis disease, medical risk factors, behavioural risk factors

## Abstract

Background: Tuberculosis (TB) remains a major global health threat, particularly in low- and middle-income countries, with TB infection (TBI) serving as the primary source of TB disease. While HIV infection has long been recognised as a major risk factor for TB progression, the rise of Non-Communicable Diseases (NCDs), which may exert immunosuppressive effects, further compounded by their treatment, contributes to increased TB susceptibility. This scoping review synthesises evidence from systematic reviews on medical and behavioural risk factors for TBI progression to TB disease, for both asymptomatic and symptomatic disease. Methods: A preliminary literature search was conducted on 11 January 2025, in PUBMED using the keywords “tuberculosis,” “asymptomatic or subclinical tuberculosis” “risk factors,” and “systematic review” followed by targeted reviews on the identified medical and behavioural risk factors for TB infection progression to TB disease. Results: A total of 25 systematic reviews were included. Medical risk factors for progression from TB infection to TB disease included diabetes mellitus (DM), chronic kidney disease (CKD), chronic obstructive pulmonary disease (COPD), undernutrition (including iron and vitamin D deficiency), cancer—particularly haematological malignancies—and immunosuppressive therapies (TNF-α inhibitors and glucocorticoids). Iron and vitamin D deficiency, particularly severe deficiency, is linked to increased TB risk, especially among people living with HIV. Behavioural risk factors, including tobacco, drug, and alcohol use, were also highlighted. Geographic variations in TB prevalence, diagnostic practices, and healthcare systems contributed to differences in risk estimates across reviews. No systematic reviews were identified that examined risk factors for asymptomatic TB. Conclusions: The convergence of TB with NCDs, compounded by immunosuppressive therapies, poses a public health challenge in high TB burden settings. Effective TB prevention requires targeted screening, along with enhanced management of these NCDs. Nutritional support, particularly screening and treatment of anaemia and vitamin D deficiency, may benefit individuals with TBI, comorbid NCDs, and HIV. A multidisciplinary approach, integrating behavioural interventions and tailored prevention strategies, is essential to achieving WHO’s End TB targets. Addressing the evidence gap on risk factors for asymptomatic TB is also critical to improve early detection and interrupt transmission.

## 1. Introduction

Tuberculosis (TB) remains a global health threat, with the World Health Organization (WHO) reporting that 10.7 million people developed TB in 2024 [1] and 1.23 million people died from the disease in 2023. Over the past three decades, global TB control efforts have been guided by successive WHO strategies, although progress has been uneven across regions and income groups. In 2024, Koura and Harries analyzed TB case notification rate (CNR) trends from 1995 to 2022 across 208 countries and demonstrated persistent disparities, with low-income and lower-middle-income countries consistently reporting substantially higher CNRs compared to high-income settings [2].

An estimated quarter of the global population is infected with *Mycobacterium tuberculosis* [3], which serves as the principal source for the development of TB disease. While progression from TB infection to TB disease is rare in immunocompetent individuals, it is heavily influenced by immunosenescence [4], genetic susceptibility [5], and immune dysregulation, with HIV infection being the most potent risk factor due to its severe impairment of T-cell-mediated control of *M. tuberculosis*, even with antiretroviral therapy [6]. Although the risk of progression to TB disease declines over time after *M. tuberculosis* infection, it remains possible for decades [7], especially in immunocompromised individuals. Not all individuals with bacteriologically confirmed TB report symptoms suggestive of TB during screening. Asymptomatic, or subclinical TB is often identified through screening or prevalence surveys. Recent reviews estimate that about half of those detected in national TB prevalence surveys had bacteriologically confirmed disease but reported no classic symptoms of TB, such as persistent cough [8,9].

While HIV infection has historically been the leading attributable risk factor for TB disease since it first came to global attention in the 1980s, recent WHO estimates indicate a shift towards a growing contribution of non-communicable and behavioural determinants. In 2024, undernutrition accounted for approximately 1.0 million incident TB cases globally, followed by diabetes (~0.9 million), alcohol use disorders (~0.8 million), and smoking (~0.7 million), compared with approximately 0.6 million cases attributable to HIV infection [1].

The rise of non-communicable diseases (NCDs) and their immunosuppressive effects, compounded by their treatments, increases TB susceptibility, and this is increasingly recognised as a key driver of the global TB burden [10]. In 2021, there were 529 million people living with diabetes mellitus (DM) worldwide and its prevalence is increasing worldwide [11], with 80% of cases occurring in low- and middle-income countries (LMICs) [12]. Similarly, cancer incidence in LMICs is burgeoning due to increased life expectancy, expanding urbanisation and lifestyle changes [13]. With chronic kidney disease (CKD) affecting 10% of the global population [14], with 59% of cases concentrated in LMICs, CKD is also a growing concern, driven by an aging population, and limited access to healthcare [15]. Furthermore, by 2050, the number of people with chronic obstructive pulmonary disease (COPD) in LMICs is expected to be more than double that of high-income countries (HICs) [16], further compounding the dual burden of NCDs and TB.

This evolving epidemiological landscape has important implications for TB control. These conditions contribute to immune dysregulation and may increase the likelihood of progression from TB infection to TB disease. Better characterisation of medical and behavioural determinants of TB progression is therefore essential.

Preventive therapies, such as isoniazid and rifampicin, have demonstrated efficacy in reducing the risk of progression to TB disease. To attain the World Health Organization (WHO)’s “End TB Strategy”, which aims to reduce TB incidence by 90% by 2035, a deeper understanding of high-risk populations susceptible to progress from TBI to TB disease is essential. This scoping review aims to map and synthesise the current evidence on medical and behavioural risk factors for TBI progression to TB disease. This review constitutes the third paper in a structured series examining the natural history of tuberculosis.

## 2. Materials and Methods

### 2.1. Search Strategy and PEOS Questions

A general literature search was conducted on 3 January 2025, to identify medical and behavioural risk factors associated with the progression from TBI to TB disease. The initial search in the PUBMED database used the following search string: (“tuberculosis” OR “subclinical tuberculosis” OR “asymptomatic Tuberculosis”) AND (“risk factors”) AND (“systematic review” OR “meta-analysis”). Based on the identified risk factors, a targeted review was conducted on the specific factors that influence TB progression. Systematic reviews published between January 2000 and January 2025 were included to ensure updated and emerging evidence. Systematic reviews focusing exclusively on the prevalence or incidence of overlapping co-infections or TB prevalence or TB incidence were excluded. We registered our protocol with OSF https://osf.io/gn74s/overview (Accessed on 22 December 2024).

The primary research question guiding this scoping review was: What are the medical and behavioural risk factors for TBI progression to TB disease? To structure the review, the following PEOS question was formulated to systematically scope relevant literature.

*Population:* Individuals of all ages and groups at risk for active TB (TB disease), including both symptomatic or asymptomatic TB.
*Exposure:* Any medical/behavioural risk factors for progressing from TB infection to active TB (TB disease).
*Outcome:* Active TB or TB disease; asymptomatic or subclinical TB.
*Study Design:* Systematic reviews published within the specified timeframe (2000–11 January 2025).

This study was designed as a scoping review of systematic reviews. Unlike an overview (umbrella review), which aims to synthesize and compare effect estimates across reviews, our objective was to map the breadth of evidence available on medical and behavioural determinants for progression to TB disease, characterize the magnitude and consistency of reported associations, and identify evidence gaps. The scoping methodology was therefore considered appropriate to provide a structured mapping of the existing review-level evidence [17].

### 2.2. Inclusion and Exclusion Criteria

Systematic reviews from 2000 meeting the eligibility criteria were screened based on their titles and abstracts, followed by a full-text review to confirm relevance. Original research, commentaries, editorials, and other types of literature, and systematic reviews which only included studies before 2000 were excluded.

Although our conceptual framework focuses on determinants of progression from TB infection to TB disease, we included systematic reviews reporting risk estimates for active TB disease. In most primary studies, prior TB infection status is not microbiologically documented. Therefore, reported risk factors for active TB disease were interpreted as proxies for determinants of progression from latent infection at the population level, recognising this inherent limitation.

### 2.3. Data Extraction, Synthesis, and Reporting

Data from the included studies were independently extracted by two reviewers using these predefined criteria to ensure reliability and minimise bias. Any discrepancies were resolved through discussion and consensus. Key findings were synthesized into categories which emerged organically from the data based on the reviewed literature. The scoping review adhered to PRISMA-ScR (Preferred Reporting Items for Systematic Reviews and Meta-Analyses, Extension for Scoping Reviews) guidelines [18]. The PRISMA-ScR checklist is available in in the Supplementary Materials Table S1.

### 2.4. Use of Non-Stigmatising Language

We have adopted non-stigmatising and person-centered terminology throughout this manuscript when referring to TB and individuals affected by it. Terms such as “people with TB,” have been used instead of “TB patients” or “TB cases”. We also use “TB infection” instead of “latent TB infection” and refer to “TB disease” rather than “active TB,” except where legacy terms are needed for clarity in cited literature. This linguistic approach aims to reduce stigma, promote respect and dignity, and reflect the evolving norms in global TB research and practice [19].

## 3. Results

### 3.1. PRISMA Flow Diagram and Systematic Review Characteristics

**Figure 1:**
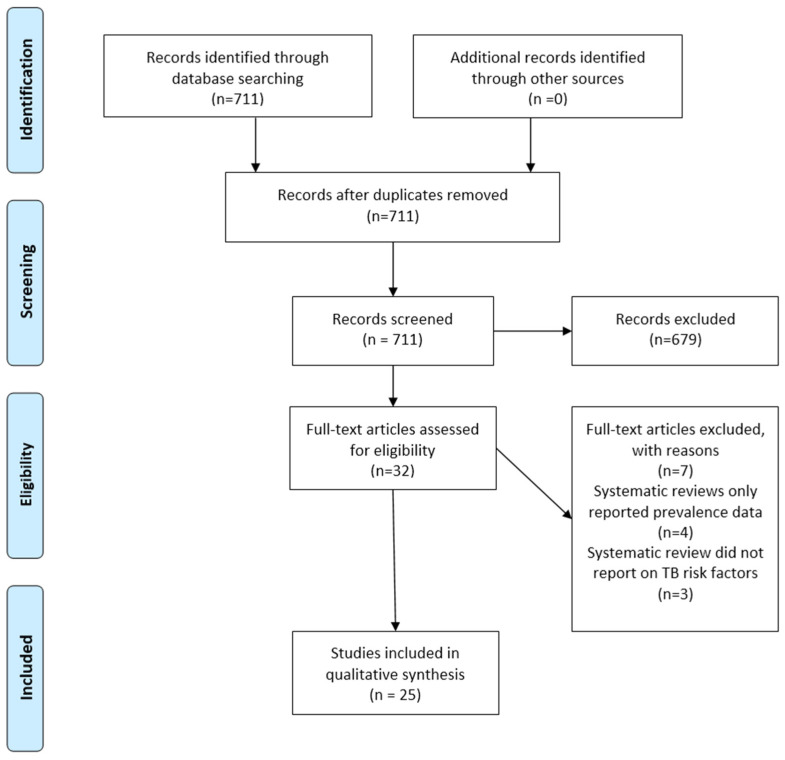
PRISMA Flow Diagram.

A general literature search identified DM, CKD, tumour necrosis factor-alpha (TNF-α) mediated diseases, malnutrition, and COPD, as potential risk factors for TBI progression, for which relevant systematic reviews were retrieved. Additionally, behavioural risk factors, including tobacco use, drug use, and alcohol consumption, were explored, with systematic reviews available for tobacco and alcohol use. Out of a total of 711 systematic reviews identified, 25 were retained (see Figure 1 for the PRISMA flow diagram and Table 1. for the Systematic Review Characteristics). To facilitate comparison across conditions, we provide a structured comparative synthesis of key risk factors, including approximate ranges of reported associations, consistency across reviews [Yes/No/Not Applicable (NA, if only one review)], and potential programmatic implications (Table 2).

### 3.2. Medical Risk Factors

#### 3.2.1. Diabetes Mellitus

The precise mechanisms linking diabetes mellitus (DM) and increased susceptibility to TB are not fully elucidated; however, chronic hyperglycaemia has been associated with impaired immune cell function and reduced host defence capacity, potentially increasing the risk of TB disease [45].

Four systematic reviews confirm this link, with risk varying by level of glycaemic control, study design, and regional TB burden.

Al-Rifai et al. (2017) [20] reported a significant association between DM and TB disease, systematically reviewing 44 studies involving 58,468,404 participants across 16 countries. The pooled adjusted risk of TB disease was highest in prospective studies (3.59-fold; 95% CI: 2.25–5.73), followed by case–control studies (2.09-fold; 95% CI: 1.71–2.55), and lowest in retrospective studies (1.55-fold; 95% CI: 1.39–1.72). The association was more pronounced in LMICs (3.16-fold vs. 1.73-fold in HICs), particularly in regions with high TB incidence (>50 cases per 100,000 person-years). Similarly, Hayashi et al. (2018) [21] reviewed 14 studies (22.6 million participants), finding that DM increases the risk of TB disease by 1.5 times (95% CI: 1.28–1.76), with greater risk in those with poor glycaemic control.

Foe-Essomba et al. (2021) [22] further analysed 47 observational studies involving 503,760 cases and 3,596,845 controls to evaluate the association between DM and TB, while accounting for a broader range of confounders, including age, gender, alcohol use, smoking, and comorbidities. The pooled analysis found that DM was associated with a significantly increased risk of TB (OR: 2.3, 95% CI: 2.0–2.7). Sensitivity analyses demonstrated that the DM-TB association remained robust even when studies with balanced confounder distributions were included. Jeon et al. (2022) [23] reinforced these findings, showing a RR of 3.11 (95% CI: 2.27–4.26) for TB in people with DM across 13 studies, emphasising regional disparities and the need for targeted TB interventions among people with DM, with subgroup analysis revealing stronger associations outside of North America.

#### 3.2.2. Malnutrition, Vitamin D Deficiency and Anaemia

Malnutrition, particularly prevalent in LMICs with high TB rates, weakens host immune responses, including cell-mediated immunity, and may impair the body’s ability to contain TB infection, thereby increasing the likelihood of progression to TB disease.

Three systematic reviews examined the impact of malnutrition on TB risk.

Franco et al. (2024) [24] reviewed 51 cohort studies with over 27 million participants to evaluate the role of undernutrition in increasing the risk of TB. The analysis showed that undernutrition doubled the risk of TB in the short term (hazard ratio [HR]: 2.23; 95% CI: 1.83–2.72). For follow-ups under 10 years, the risk remained consistent (HR 2.02, 95% CI: 1.74–2.34), while at 10+ years, the risk surged (HR 12.43, 95% CI: 5.74–26.91) [24]. In a meta-analysis (3544 participants), Aibana et al. (2019) [25] further showed that vitamin D deficiency increased TB risk (aOR 1.48, 95% CI 1.04–2.10, *p* = 0.03), with severe deficiency showing a stronger trend. Among 1576 HIV-positive individuals, vitamin D deficiency doubled TB risk (aOR 2.18, 95% CI 1.22–3.90). Results suggest a dose-dependent TB risk, especially in HIV-positive individuals. Furthermore, Gelaw et al. (2021) [26] in a systematic review and meta-analysis of 17 studies (*n* = 215,294) found that anaemia significantly increased the risk of TB disease. Anaemic individuals had higher odds (OR 3.56, 95% CI: 2.53–5.01) and hazard ratios (HR 2.01, 95% CI: 1.70–2.37) of developing TB compared to non-anaemic individuals, with risk rising with anaemia severity.

#### 3.2.3. Cancer

Cancer is associated with immunosuppression, caused by the malignancy itself or its treatment, increasing the risk of bacterial infections such as TB.

Two systematic reviews suggested this association.

Cheng et al. (2016) [27] explored TB incidence in 324,041 cancer patients through a meta-analysis of 23 studies. The highest TB incidence was observed in patients with hematologic cancers (219 per 100,000), head and neck cancers (143 per 100,000), and lung cancers (83 per 100,000), with corresponding incidence rate ratios (IRRs) of 26, 16, and 9, respectively. In contrast, patients with breast and other solid cancers had the lowest incidence (38 per 100,000; IRR = 4). Dobler et al. (2017) [28] analysed 13 studies with 921,464 cancer patients to assess the risk of TB in this population. Comparing incidence rates of TB rather than cumulative incidence or prevalence, the study found that the incidence rate ratio (IRR) for TB in cancer patients was 2.61 (95% CI: 2.12–3.22). The IRR was also higher in patients with haematological cancers (3.53, 95% CI: 1.63–7.64) compared to those with solid cancers (2.25, 95% CI: 1.96–2.58). Notably, children with either haematological or solid cancers had the highest IRR (16.82, 95% CI: 8.81–32.12).

#### 3.2.4. Chronic Kidney Disease (CKD)

Due to impaired immunity, people with CKD are at increased risk of infections [46]. Two systematic reviews assess this association.

Luczynski et al. (2023) [29] evaluated the TB risk in individuals with CKD stages 3–5, excluding those with kidney failure or undergoing kidney replacement therapy. The analysis of five observational studies revealed a 57% increased risk of TB in CKD patients compared to those without CKD (adjusted HR: 1.57, 95% CI: 1.22–2.03). Advanced CKD stages (4–5) were associated with the highest TB risk, showing a 3.63-fold increased IRR (95% CI: 2.25–5.86). Furthermore, Al Efraji et al. (2015) [30] identified a significantly higher risk of TB disease in end-stage renal disease patients, with dialysis patients having a 3.62-fold increased risk (95% CI: 1.79–7.33) and renal transplant recipients within end-stage renal disease populations having an 11.35-fold increased risk compared to the general population (unadjusted risk ratio: 11.35; 95% CI: 2.97–43.41). The review reported these estimates according to renal replacement therapy modality, but did not further analyze the mechanisms underlying the higher risk observed in transplant recipients.

#### 3.2.5. Tumor Necrosis Factor-Alpha (TNF-α) Inhibitors

TNF-α is crucial for containing *M. tuberculosis* infection by maintaining granuloma integrity and mediating host defences. However, TNF-α inhibitors, used to treat conditions like multiple sclerosis (MS), rheumatoid arthritis (RA), inflammatory bowel disease (IBD), and Systemic Lupus Erythematosus (SLE), impair granuloma formation [47], increasing the risk of progression from TBI to TB disease.

Six systematic reviews suggested an association between TNF-α inhibitors and an increased risk of TB across various immune-mediated diseases.

Wu et al. (2022) [31] reviewed 35 studies on 46,327 SLE patients, finding a non-significant increase in TB incidence of 1.16 per 100 person-years (95% CI: 0.69–1.93). Higher TB risk was observed in Africa (*p* < 0.01), high TB-burden areas (*p* = 0.03), and among those on ≥20 mg daily glucocorticoids, highlighting the need for targeted TB screening and prevention in at-risk populations (*p* = 0.04). Pego-Reigoso et al. (2021) [32] assessed 11 cohort studies to determine infection risks in SLE patients. The pooled RR for TB in SLE patients was significantly increased compared to the general population/healthy controls (RR 6.11; 95% CI 3.61–10.33), with sensitivity analyses confirming the robustness of the findings, emphasising the need for targeted TB prevention strategies in SLE populations.

For MS, Dantas et al. (2021) [33] reviewed 14 studies on MS treatments and TBI reactivation, finding that immunosuppressive therapies, especially disease-modifying therapies and biologics, significantly increase the risk. Most studies recommended pre-treatment TB screening for people with MS, particularly in high-burden areas, highlighting the need for improved screening protocols for at-risk populations. Souto et al. (2014) [34], who further reviewed 100 RCTs (75,000 patients) and 63 long term extension studies (>80,000 patient-years), assessed TB risk in immune-mediated inflammatory disease patients treated with biologics and tofacitinib. TNF-α inhibitors were linked to 31 people with TB, with a non-significant OR of 1.92 (95% CI: 0.91–4.03) in RCTs. Long term extension studies reported that TB risk was highest with TNF monoclonal antibodies in those with RA (RR: 307.71 per 100,000; 95% CI: 184.79–454.93), and this was strongly influenced by regional TB prevalence. Similarly, Ai et al. (2015) [35] reviewed 50 RCTs and 13 cohort studies and found that TNF-α antagonists significantly increased TB risk in those with RA (RR 4.03, 95% CI 2.36–6.88).

Combination therapy carried a higher risk compared with monotherapy, though anti-TNF monotherapy also showed increased TB risk. Similarly, Zhang et al. (2017) [36] assessed the risk of TB in patients receiving TNF-α antagonists among the 7912 patients treated with TNF-α antagonists, yielding an overall OR of 1.94 (95% CI: 1.10–3.44, *p* = 0.02). Subgroup analysis revealed that patients with RA had an even higher risk when treated with TNF-α antagonists (OR: 2.29, 95% CI: 1.09–4.78, *p* = 0.03) and 0.57% of patients on TNF-α antagonists developed TB, compared to 0.08% in control groups (OR = 1.94, 95% CI: 1.10–3.44, *p* = 0.02).

#### 3.2.6. Chronic Obstructive Pulmonary Disease (COPD)

Chronic respiratory diseases, such as COPD, also increase TB risk due to impaired lung function and heightened susceptibility to infections. Inhaled corticosteroids (ICS), central to asthma management and severe COPD management, control inflammation but suppress immunity, thereby further increasing infection risk [48].

Two systematic reviews suggested an association between COPD and increased TB risk, particularly with ICS use.

Dong et al. (2014) [37] analysed 25 RCTs with 22,898 persons with COPD for TB risk and 23,616 for influenza, finding that ICS use doubled the risk of TB (Peto OR: 2.29; 95% CI: 1.04–5.03), with higher risk in TB-endemic regions. Similarly, Castellana et al. (2019) [38] reviewed nine studies with 36,351 ICS users and 147,171 non-users, showing a 46% increased TB risk with ICS use (OR: 1.46, 95% CI: 1.06–2.01, *p* = 0.02), particularly in current users. The risk was even higher in those not using oral corticosteroids (OR: 1.63, 95% CI: 1.05–2.52, *p* = 0.03).

### 3.3. Behavioural Risk Factors

Behaviours such as smoking and alcohol consumption are critical modifiable risk factors that influence TB progression. Smoking may impair mucociliary clearance and alter host immune responses, while alcohol consumption may be associated with immune dysregulation and increased susceptibility to infections [49].

Six systematic reviews have highlighted the significant role of smoking (4) and alcohol (2) in increasing TB risk.

Bates et al. (2007) [39] analysed 24 epidemiologic studies, finding that active smoking significantly increased TB risk (RR 2.33–2.66), while Slama et al. (2007) [40] reviewing 42 studies found strong evidence linking smoking to TB and moderate evidence for second-hand smoke exposure. Dogar et al. (2015) [41] examined 12 studies on second-hand smoke exposure in non-smokers, reporting a higher TB risk (RR 1.59, 95% CI: 1.11–2.27), with variability due to differing diagnostic criteria. In children, Patra et al. (2015) [42] showed that second-hand smoke exposure increased the risk of TB disease, with children having a more than three-fold higher risk (RR 3.41, 95% CI 1.81–6.45), compared to a smaller increase in adults (RR 1.32, 95% CI 1.04–1.68). Strong adjusted exposure-response relationships were seen in young children, those exposed through any parent, and those in crowded households.

Alcohol consumption has been shown to increase TB risk. Lönnroth et al. (2008) [43] reviewed 21 observational studies, finding that alcohol consumption of ≥40 g per day or an alcohol use disorder increased the risk of TB disease (RR 2.94, 95% CI: 1.89–4.59), likely due to immune suppression and higher exposure risk linked to alcohol-related behaviours. Similarly, Simou et al. (2018) [44] reviewed 49 studies, showing that alcohol consumption, even at low levels, raised TB risk (OR 1.90, 95% CI: 1.63–2.23). Further sensitivity and hazard ratio analyses confirmed a slightly lower but still elevated risk, with a 12% increase in TB risk per 10–20 g of daily alcohol intake.

## 4. Discussion

### 4.1. Summary of Results

This scoping review identified multiple medical and behavioural determinants associated with progression from TB infection to TB disease. Rather than acting in isolation, these conditions form a heterogeneous but patterned landscape of risk, with substantial variation in the magnitude of association across exposures.

Across conditions, the magnitude of association varied considerably. The highest relative risks were observed among transplant recipients, patients receiving TNF-α inhibitors, and individuals with haematological malignancies. Moderate but consistently elevated risks were seen in people with diabetes, CKD, and undernutrition. Behavioural determinants such as smoking and alcohol use showed more moderate relative risks but have substantial population-level impact due to their high prevalence. Importantly, while some conditions confer high individual-level risk, others such as diabetes and undernutrition contribute more significantly at the population level because of their widespread distribution. This distinction is critical for prioritizing TB screening and preventive strategies in different epidemiological settings.

Several cross-cutting themes emerge from this synthesis. Many of the identified conditions are characterised by some degree of immune dysregulation or immunosuppression, whether related to metabolic dysfunction, micronutrient deficiency, malignancy, organ failure, or therapeutic immune modulation. Although the included systematic reviews primarily reported epidemiological associations rather than detailed mechanistic pathways, impaired host immune responses—particularly affecting cell-mediated immunity and the maintenance of granuloma integrity—appear to represent a common underlying pathway across several conditions.

Contextual variation was also evident. Stronger associations were frequently reported in low- and middle-income countries, likely reflecting a higher background TB burden, differences in healthcare access, comorbidity patterns, and broader socioeconomic determinants. Finally, no systematic review specifically addressing risk factors for asymptomatic TB progression was identified, highlighting an important gap in the current evidence base. 

### 4.2. Public Health Impact

#### 4.2.1. Diabetes and Contextual Heterogeneity

The public health impact of the synergistic interactions is vast with the global DM burden in LMICs projected to increase substantially, driven by aging populations and the rising prevalence of obesity and tobacco use [50]. The stronger associations observed in LMICs compared with HICs may reflect several contextual factors. Higher background TB incidence increases the probability of exposure and progression among people with diabetes. In addition, differences in glycaemic control, delayed diagnosis, limited access to integrated diabetes and TB care, and underdiagnosis of both conditions may amplify risk estimates in resource-constrained settings. Socioeconomic determinants, nutritional status, and coexisting infections may further modify the observed associations. These contextual differences underscore the importance of adapting TB screening and prevention strategies to local epidemiological conditions. Due to resource constraints, targeting all individuals with DM for TBI screening and treatment may not be feasible. Given the evidence suggesting that poorer glycaemic control is associated with higher TB risk, prioritizing TB preventive interventions among people with diabetes with suboptimal glycaemic control may be more feasible than targeting all individuals with diabetes in resource-constrained settings. The thresholds corresponding to standard diagnostic criteria for diabetes (fasting plasma glucose ≥ 126 mg/dL or HbA1c ≥ 6.5%) are used solely to define diabetes status and should not be interpreted as TB-specific risk thresholds. Further studies are needed to determine clinically meaningful glycaemic thresholds relevant for TB risk stratification.

#### 4.2.2. Cancer and Immunosuppression

Current evidence indicates that people with cancer, particularly those with haematological, head and neck, or lung cancers, have a significantly elevated risk of developing TB disease due to the immunosuppressive effects of both the malignancy itself and its treatments. Among these groups, children with haematological malignancies or solid tumours in high TB incidence settings are at the highest risk and may benefit from systematic TBI screening. In adults with cancer, TB risk appears to be highest during periods of active immunosuppression, particularly in patients receiving intensive chemotherapy or other immunomodulatory treatments. However, the balance between benefits and risks of routine TBI screening in adults with solid tumours remains uncertain and may depend on background TB incidence, comorbidity profile, and individual risk factors. While concerns regarding hepatotoxicity from preventive therapy have historically influenced recommendations, the availability of shorter and better-tolerated regimens may modify future risk–benefit assessments. Screening strategies should therefore be risk-based and contextualised rather than based solely on cancer type or survivorship status.

#### 4.2.3. Chronic Kidney Disease

While current WHO guidelines prioritise TB screening for patients on dialysis or those who have undergone kidney transplantation [51], emerging evidence suggests that earlier-stage CKD patients may also face an elevated TB risk. However, its magnitude relative to other high-risk groups remains unclear, underscoring the need to assess the feasibility and cost-effectiveness of broader screening. Expanding screening must be balanced against other priorities, and the current absence of clear risk thresholds for CKD progression and TB reactivation raises concerns about overtreatment, nephrotoxicity, and missed prevention opportunities.

#### 4.2.4. Therapeutic Immunosuppression and COPD

Furthermore, the growing use of TNF-α inhibitors in autoimmune diseases and ICS in COPD as risk factors for TB reactivation, particularly when combined with other immunosuppressive treatments, is a cause for concern. This calls for more rigorous monitoring for TB in these populations in higher TB-burden settings. These findings also underscore the need for cost-effectiveness analyses to guide TBI screening before starting TNF-α inhibitors, particularly in regions with high TB prevalence and among migrants from these endemic settings.

#### 4.2.5. Malnutrition and Micronutrient Deficiencies

Moreover, malnutrition, especially undernutrition, accelerates progression from TBI to TB disease, a major concern in LMICs where it coexists with rising NCD burdens like DM and CKD. Therefore, integrating nutritional support into TB prevention strategies for individuals with TBI and comorbid NCDs is crucial. Emerging evidence highlights the dose-dependent role of iron deficiency and vitamin D deficiency in increasing TB risk, particularly in HIV-positive individuals, emphasising the need for nutritional screening and deficiency management, in TB prevention for those with TBI and comorbid NCDs.

#### 4.2.6. Smoking and Multimorbidity

Smoking not only increases the risk of TB, but is also linked to the development of CKD [42,52], DM [53], COPD [54], and autoimmune diseases [55]. These conditions interact synergistically, exacerbating the risk of TB progression, particularly in an HIV-infected population. This quadripartite relationship between smoking, chronic diseases, HIV infection and TB highlights the need to design a multi-sectoral approach, combining targeted prevention efforts, particularly in regions with a high dual burden of TB and HIV, as well as among immigrant populations in HICs from high TB-prevalence countries in LMICs.

### 4.3. Research Gaps

Optimising TB prevention requires cost-effectiveness studies to define immunosuppression thresholds, HbA1c levels, and CKD stages warranting intervention, particularly in high TB-burden regions with limited DM and CKD care.

There is a need for well-designed, large-scale RCTs to assess the efficacy and optimal dosage of vitamin D supplementation in reducing TB risk, particularly among high-risk populations, including those with HIV and comorbid NCD populations.

No systematic reviews to date have examined risk factors specific to asymptomatic TB. This lack of evidence limits the ability to identify high-risk populations and hinders the development of targeted screening strategies beyond symptom-based approaches. As a result, many infectious but asymptomatic individuals remain detected, sustaining transmission in the community and undermining TB control efforts.

In LMICs, further research is needed to quantify TB risk in individuals with early-stage CKD and identify key stages of heightened TB susceptibility. Similarly, the immunosuppressive effects of malignancies and their treatments, especially in haematological cancers, require further investigation to determine thresholds for TB reactivation risk: this is especially important in LMICs with rising cancer rates and higher HIV burdens. This also applies to TNF-mediated inflammatory diseases.

High-quality, large-scale studies are essential to assess the role of undernutrition in TB and inform strategies for integrating nutritional support into TB control programmes for better prevention.

Advanced analytical approaches, including machine learning and mathematical modelling, may help address several of the evidence gaps identified in this review. In particular, these methods could be used to model joint risk profiles, explore interaction effects among coexisting exposures (e.g., diabetes, smoking, HIV), and improve individual-level risk stratification in settings with complex multimorbidity patterns. Predictive modelling approaches may also support the identification of individuals at risk of asymptomatic or subclinical TB, thereby informing more targeted screening strategies. However, such approaches require high-quality longitudinal data and careful validation to avoid bias and overfitting.

Most included systematic reviews evaluated single exposures in isolation, and few formally assessed combined or interaction effects between risk factors. Evidence on additive or multiplicative risks—for example, the combined impact of diabetes and HIV, smoking and chronic kidney disease, or immunosuppressive therapy in metabolically compromised individuals—remains limited. Given that patients in real-world settings frequently present with multiple overlapping conditions, understanding synergistic effects is essential. Future research should prioritise studies examining multi-morbidity and interaction modelling to better inform risk stratification and screening thresholds.

An additional gap identified in this review concerns the limited reporting of dose–response relationships for behavioural risk factors. The included systematic reviews on smoking did not consistently quantify exposure intensity (e.g., pack-years or cigarettes per day), and dose–response analyses were not systematically performed. Similar concerns apply to alcohol use. Standardised reporting of exposure intensity would strengthen risk stratification and help refine screening and prevention strategies.

Importantly, no systematic review specifically addressing risk factors for asymptomatic or subclinical TB progression was identified. This is a major evidence gap, particularly as national TB prevalence surveys and community-based screening activities have repeatedly shown that a substantial proportion of bacteriologically confirmed TB can be detected in individuals without typical symptoms at the time of screening. Reliance on symptom-based algorithms alone may therefore miss a meaningful fraction of infectious TB, delaying diagnosis and allowing ongoing community transmission. As TB programmes increasingly adopt screening approaches that go beyond symptoms (e.g., systematic screening in high-risk groups, use of radiography and rapid diagnostics), the absence of evidence on determinants of asymptomatic progression limits the ability to optimise targeting, design risk-based algorithms, and allocate resources efficiently. Future research should prioritise prospective cohorts and dedicated evidence syntheses to characterise risk profiles for asymptomatic/subclinical TB and to inform screening strategies aligned with evolving WHO recommendations.

### 4.4. Strengths and Limitations

The inclusion of only systematic reviews enhances the robustness of the results, and the absence of language restrictions broadens the scope and inclusivity of the evidence base. However, a limitation may be that by focusing exclusively on systematic reviews, we may have failed to capture emerging evidence on other comorbidities. At the evidence level, systematic reviews on DM lacked representation from Sub-Saharan Africa, making the findings less easy to extrapolate. Studies on malnutrition often lacked consistent long-term follow-up, while cancer-related research was limited to high-income, low-TB-prevalence settings. CKD and autoimmune disease studies lacked standardised screening protocols, with TB risk estimates varying by disease and region. Furthermore, most studies in the reviews were observational, limiting causal associations between behavioural risk factors, NCDs, and TB. In addition, variability in controlling key confounders, such as hospitalization and immigration status, may have led to residual confounding and biased estimates.

A conceptual limitation relates to the fact that most included systematic reviews reported risk or incidence of active TB without distinguishing between progression from pre-existing TB infection, reactivation, or reinfection. As this scoping review synthesised evidence as defined and reported in the included reviews, it was not possible to systematically distinguish between progression, reactivation, and reinfection mechanisms underlying incident TB cases. This may introduce conceptual imprecision, particularly in high-transmission settings where reinfection may contribute to disease occurrence.

Finally, this scoping review relied on PubMed as the primary database, which may have resulted in the omission of relevant systematic reviews indexed exclusively in other databases, particularly those covering environmental or social science literature.

## 5. Conclusions

The convergence of TB with CKD, DM, COPD, autoimmune diseases, and cancer presents a public health challenge, particularly in LMICs with moderate to high TB burdens and overlapping prevalence of HIV infection. This challenge is also compounded through the use of immunosuppressive treatments, such as TNF-α inhibitors and corticosteroids, which increase the risk of TB reactivation. Evidence links TB risk to NCDs, smoking, and alcohol use, but more data and cost-effectiveness studies across diverse TB burden settings are needed for refinement. Until then, clinicians should maintain a high index of suspicion for TB, particularly in vulnerable groups like those in LMICs and migrants from high-burden areas, while weighing the associated risks of hepatotoxicity and nephrotoxicity with TB preventive treatment. Malnutrition, particularly undernutrition, accelerates TBI progression to TB disease, highlighting the need for integrated nutritional support, including iron and vitamin D supplementation, in TB prevention among individuals with comorbid NCDs, and HIV infection.

Moreover, strengthening behavioural interventions, such as smoking cessation and alcohol use prevention programs, is crucial especially in high-risk populations. Importantly, the absence of robust evidence on risk factors for asymptomatic TB further limits early detection, allowing ongoing community transmission. To meet WHO’s TB End TB strategy by 2035, a multidisciplinary approach for public health interventions is vital for addressing the quadruple burden of TB, NCDs, malnutrition, and HIV and ultimately global health.

## Figures and Tables

**Table 1:** Systematic review characteristics included in the current scoping review.

Author of Systematic Review	Date Range of Eligible Studies	Number of Studies	Objective	Methodology Assessment	Conclusion
Al Rifai et al. (2017) [20]	1995–2014	44 eligible studies were included, which consisted of 58,468,404 subjects from 16 countries.	Provide a summary estimate of the association between DM and active TB	36/44 had low risk of bias using the Cochrane adapted tool	Meta-analysis revealed that DM patients had a 3.59-fold increased risk of active TB in prospective studies, 1.55-fold in retrospective studies, and 2.09-fold in case–control studies. The association was stronger in low/middle-income countries (3.16-fold) compared to high-income countries (1.73-fold) and varied by geographical region and TB incidence.
Hayashi et al. [21]	1992–2017	14 studies (8 cohort and 6 case–control) involving 22,616,623 participants	Assess the risk of active TB in people with DM and the factors associated with this risk.	Newcastle-Ottawa scale: 1–9 (score of studies: 6–9)	DM increases TB risk by 1.5 times (95% CI 1.28–1.76), with moderate heterogeneity (I^2^ = 44%), particularly among those with poor glycaemic control. Strengthening glycaemic management may help mitigate TB risk, underscoring the need for an integrated approach to address the dual burden of DM and TB.
Foe-Essomba et al. (2021) [22]	2006–2019	47 observational studies (503,760 cases and 3,596,845 controls). No studies from Latin America	Determine whether DM is associated with an increased risk of developing TB with sensitivity analyses incorporating a wider range of confounders	Using the Joanna Briggs Institute scale, overall, the includes had a low risk of bias 32/49.	The pooled odds ratio (OR = 2.3, 95% CI = [2.0–2.7], I^2^ = 94.2%) confirmed a significant association between DM and TB risk, consistent across study designs (cohort: OR = 2.0, case–control: OR = 2.4, cross-sectional: OR = 2.5). Sensitivity analysis further validated these findings. The substantial heterogeneity observed was primarily due to geographic variations
Jeon et al. (2008) [23]	1995–2007	13 observational studies with 1,786,212 participants and 17,698 TB cases	Explore the association of DM and TB to summarize the existing evidence and to assess methodological quality of the studies.	None carried out	The random effects meta-analysis of cohort studies showed a relative risk of 3.11 (95% CI 2.27–4.26), while case–control studies exhibited greater variability (odds ratios ranged from 1.16 to 7.83). Subgroup analysis indicated higher effect estimates in studies outside of North America. non-North American studies.
Franco et al. (2024) [24]	1971–2021	51 cohort with 27 million participants. geographical representation of the places with the highest burden of TB is only partially represented in the current body of evidence. There were no large studies in the Americas	Determine the prognostic value of undernutrition in the general population of adults, adolescents, and children for predicting TB over any time period. HIC and LMICs (including SSA)	Cochrane methodology and the Quality In Prognosis Studies tool to assess the risk of bias of the studies. Certainty of evidence: very low-moderate	The median follow-up time was 3.5 years, with most studies reporting adjusted hazard ratios (HR). Undernutrition may increase TB risk (HR 2.23, 95% CI 1.83 to 2.72) with moderate certainty, but the evidence is low for longer follow-up (>10 years). The odds ratio and risk ratio estimates suggest undernutrition may increase TB risk, but the evidence is uncertain or low quality. Policies addressing undernutrition are critical for controlling TB globally. Subgroup analysis of studies on HIV participants versus the general population showed no significant differences in estimates. Further high-quality studies, especially including children and adolescents, are needed
Aibana et al. (2019) [25]	2013–2016	7 studies in the one-stage individual-participant data meta-analysis (3544 participants). Europe, Sub Saharan Africa, Southeast Asia, Latin America	Assess the impact of baseline vitamins D levels on TB disease risk.	Newcastle-Ottawa quality assessment for non-randomised studies (1–9 scale). Score was 7–9) good.	Vitamin D deficiency increased TB risk (aOR 1.48, 95% CI 1.04–2.10, *p* = 0.03), with severe deficiency showing a stronger trend (aOR 2.05, 95% CI 0.87–4.87, *p* = 0.02). Among 1576 HIV+ individuals, deficiency doubled TB risk (aOR 2.18, 95% CI 1.22–3.90, *p* = 0.01), with severe deficiency associated with an even higher, though non-significant, risk (aOR 4.28, 95% CI 0.85–21.45, *p* = 0.08). These findings suggest a dose-dependent relationship between vitamin D levels and TB risk, particularly in HIV-positive individuals
Gelaw et al. (2021) [26]	From inception to 2019	17 studies including 215,294 participants (most studies were conducted in SSA)	Determine whether anaemia is a risk factor for TB	Joanna Brigg Institute criteria (−8 score) Not reported	Anaemia was associated with a significantly increased risk of tuberculosis, with an overall odds ratio of 3.56 (95% CI: 2.53–5.01) and a HR of 2.01 (95% CI: 1.70–2.37). The risk of TB increased with anaemia severity: HR 1.37 (95% CI: 0.92–2.05) for mild, 2.08 (95% CI: 1.14–3.79) for moderate, and 2.66 (95% CI: 1.71–4.13) for severe anaemia.
Cheng et al. (2016) [27]	23 studies (324,041 cancer patients)	Meta-analysis of 6 studies conducted in the US	Determine the incidence and RR of active TB in cancer patients compared to the general population	Newcastle-Ottawa Quality Assessment scale for Cohort studies (risk was low)	A total of 23 studies (324,041 cancer patients; 593 TB cases) were analyzed. In the U.S., TB CIR decreased 3-fold (hematologic cancers) and 6.5-fold (solid cancers) post-1980. Post-1980, the highest CIRs were in hematologic (219/100,000; IRR = 26), head and neck (143; IRR = 16), and lung cancers (83; IRR = 9), while breast and other solid cancers had the lowest (38; IRR = 4). Targeted LTBI screening and therapy may benefit patients with these high-risk cancers. US-born people with solid tumors should not be screened for latent TB due to liver damage risks from treatment, but foreign-born people might benefit from screening because newer, safer treatments are now available.
Dobler et al. (2017) [28]	1988–2016	13 studies including 921,464 patients with cancer (HIC and 1 in South Africa)	Estimate the relative incidence of TB	Newcastle-Ottawa Quality Assessment scale for Cohort studies (risk was moderate to low)	Only studies providing an incidence rate of TB, as well as a TB incidence rate in a control/general population were included. The IRR for TB was 2.61 (95% CI 2.12–3.22; I^2^ = 91%) overall, 3.53 (95% CI 1.63–7.64; I^2^ = 96%) for haematological cancers, and 2.25 (95% CI 1.96–2.58; I^2^ = 91%) for solid cancers in adults. Children with haematological or solid malignancies had the highest IRR (16.82, 95% CI 8.81–32.12; I^2^ = 79%). Systematic LTBI screening may be warranted for children, especially they originate from setting with a high TB incidence, but not adults due to differences in TB risk and life expectancy.
Luczynski et al. (2023) [29]	2020–2022	5 prospective and retrospective studies (HICs)	Estimate the pooled relative risk of TB disease in people with CKD stages 3–5 without kidney failure compared with people without CKD	Newcastle-Ottawa quality assessment scale: quality was moderate to high	Data indicate that individuals with CKD stages 3–5 have a 57% higher TB risk compared to those without CKD (aHR: 1.57; 95% CI: 1.22−2.03; I^2^ = 88%). TB risk is highest in CKD stages 4–5 (IRR: 3.63; 95% CI: 2.25−5.86; I^2^ = 89%). Further research is needed to define screening thresholds and benefits before kidney replacement therapy.
Al-Efraij et al. (2015) [30]	2005–2013	12 studies (71,374 end-stage renal disease patients and 560 TB cases) (North America, Australia, Latin America, Europe, Middle East, Asia)	Conduct a systematic review to evaluate active TB risk in CKD populations.	Newcastle-Ottawa quality assessment scale. Only 3 studies reached the quality cut-off 7 and higher considered high quality.	Meta-analysis showed a 3.62-fold higher TB rate in dialysis populations (adjusted rate ratio: 3.62; 95% CI: 1.79–7.33) and an 11.35-fold higher TB risk in transplant populations (unadjusted risk ratio: 11.35; 95% CI: 2.97–43.41) compared to the general population. The increased TB risk in end-stage renal disease persisted across study designs and renal replacement therapy modalities
Wu et al. (2022) [31]	1982–2021	35 studies (46,327 SLE patients). More than 3/4 of the studies included were conducted in endemic countries. One study from SSA.	determine the incidence and prevalence of tuberculosis (TB) in SLE patients.	Not available	TB incidence was higher in Africa and high TB-burden countries, (*p* = 0.01) in patients on ≥20 mg glucocorticoids (*p* = 0.04). A high cumulative and/or mean daily dose of GCs has been implicated as a predisposing factor for the development of TB in SLE patients. These findings underscore the need for targeted TB prevention strategies in SLE populations.
Pego-Reigosa et al. (2021) [32]	2012–2017	3 studies (England, Brazil, Singapore)	evaluate the effect of general and SLE-related factors on infection risk	Newcastle-Ottawa quality assessment scale: low risk of bias	SLE was associated with a significantly higher risk of TB compared to the general population/healthy controls (pooled RR 6.11, 95% CI 3.61–10.33). Efforts to strengthen strategies aimed at preventing infections in SLE are needed.
Dantas et al. (2021) [33]	2010–2020	14 studies	Investigate the link between MS treatments and LTBI reactivation	No assessment of risk of bias, just an exploration of limitations.	Immunosuppressive therapies, particularly DMTs and biologics, increase LTBI reactivation risk in MS patients. Pre-treatment TB screening is recommended, especially in high-burden areas, though further research is needed to address knowledge gaps and refine screening protocols.
Souto et al. (2014) [34]	Until 2013	100 RCT (75,000 patients) and 63 (Long term extension). Geographical location not specified	Assess the risk of active TB in patients with immune-mediated inflammatory diseases with biologics and tofacitinib in RCTs and LTE studies	Jadad scale and the level of evidence using the Oxford Centre for Evidence-Based Medicine Levels of Evidence. Unclear what the assessment was, although the authors stated that no asymmetries were found in the funnel plots	TNF inhibitors are associated with increased TB risk (OR 1.92, 95% CI 0.91–4.03, *p* = 0.085) compared to other biologics (e.g., rituximab, tocilizumab). In LTE studies, TB incidence rates were >40/100,000 for most biologics except rituximab, with higher rates in RA patients using anti-TNF monoclonal antibodies. TB risk was elevated in high-background TB areas. RCTs may underestimate LTBI reactivation risk.
Ai et al. (2015) [35]	1999–2014	50 RCT and 13 non-RCT (HICs) RCT unclear.	Evaluate the risk of TB infection from 5 TNF alpha antagonist drugs for patients with RA	Cochrane ROB for RCT: 6/50 had high risk of bias; 3/50 unclear risk of bias NoS: score (out of 9) 5–7	TNF-α antagonists significantly increase TB risk in RA patients (RR 4.03, 95% CI 2.36–6.88), with ETN having the lowest risk compared to IFX and ADA. LTBI prophylaxis reduces TB risk by 65% (RR 0.35, 95% CI 0.15–0.82). RCTs showed no significant difference due to short follow-up periods.
Zhang et al. (2017) [36]	1999–2014	29 RCT (11,879 patients) HIC countries	Assess the risk of TB in patients undergoing TNF-α antagonists treatment.	Evidence quality was rated as “low” (GRADE)	A meta-analysis of 29 RCTs (11,879 patients) found that TNF-α antagonists significantly increased TB risk (OR 1.94, 95% CI 1.10–3.44, *p* = 0.02), with RA patients at higher risk (OR 2.29, 95% CI 1.09–4.78, *p* = 0.03). TB incidence was 0.57% in TNF-α antagonist groups versus 0.08% in controls. Further research is required to elucidate the biological mechanisms behind this risk.
Dong et al. (2014) [37]	2011–2013	5 studies using chart review, claim database (22,898 patients) HIC	Assess the use of inhaled corticosteroids in patients with CODP and the risk of TB and influenza	All studies had a low risk of bias	Inhaled corticosteroids (ICS) treatment was associated with a significantly higher risk of TB (Peto OR, 2.29; 95% CI, 1.04–5.03). The number needed to harm for an additional TB event was lower in COPD patients treated with ICSs in endemic areas (909) compared to nonendemic areas (1667). The findings raise safety concerns regarding the TB risk associated with ICS use in COPD patients, particularly in endemic areas.
Castellana et al. (2019) [38]	2010–2017 HIC	9 studies; 36,351 patients were prescribed ICS, and 147,171 were not.	Evaluate the effects of inhaled corticosteroids (ICS) on the risk of TB in patients with obstructive lung diseases.	National Heart, Lung, and Blood Institute Quality Assessment Tools 8 to 10/12 and 11/14	ICS use was associated with an increased TB risk (OR = 1.46; 95% CI, 1.06–2.01), but only 0.49% of TB cases were attributed to ICS. The risk was more pronounced in patients not on oral corticosteroids (OR = 1.63; 95% CI, 1.05–2.52). While ICS increases TB risk, its overall contribution is limited, and no population-based interventions are needed. Risk should be assessed individually, particularly in high-risk patients.
Bates et al. (2007) [39]	1953–2005	24 studies (Europe, North America, South east Asia, sub Saharan Africa)	Quantify the relationship between active tobacco smoking and TB pulmonary disease	No risk of bias assessment was performed	Smoking is a significant risk factor for both TB infection (RR 1.73, 95% CI: 1.46–2.04) and TB disease (RR ranging from 2.33 to 2.66), suggesting a 1.4 to 1.6-fold increased risk of disease development in individuals with TB infection.
Slama et al. (2007) [40]	1954–2005	42 studies (North America, Sub Saharan Africa, Europe, Southeast Asia)	Assess the strength of evidence in for an association between smoking and passive exposure to tobacco smoke and various manifestations and outcomes of TB	Strength of evidence for TB disease was assessed as strong, according to author’s criteria.	The evidence strongly supports a causal association between smoking and TB disease, with moderate evidence for a link between second-hand smoke exposure and TB disease, as well as smoking and retreatment TB disease
Dogar et al. (2015) [41]	1996–2014	12 studies (Europe, North America, South east Asia, sub Saharan Africa)	Evaluate the association between second-hand smoke (SHS) and the risk of acquiring and worsening of TB in non-smokers.	Newcastle-Ottawa scoring system: poor to good	Exposure to SHS was found to be statistically significantly associated (RR 1.59, 95% CI 1.11–2.27) with the risk of TB disease. There was significant heterogeneity (I^2^ = 77%, *p* = 0.0006) between studies’ results, which was sourced to the internal characteristics of the studies rather than combining different study designs.
Patra et al. (2015) [42]	1996–2014	18 studies (30,757 children and 44,432 adult non-smokers) Europe, South-east Asia, SSA, Americas	Investigate the role of second-hand smoke (SHS) exposure as a risk factor for TB among children and adults.	Newcastle-Ottawa scoring system: poor to good	SHS exposure was linked to a higher risk of LTBI and active TB, with children showing a significantly greater risk of active TB (RR 3.41) than adults (RR 1.32). The risk was higher in younger children, those exposed to any parent, and those in crowded households. While associations remained significant after adjusting for age, biomass fuel use, and TB contact, the link with LTBI weakened after adjusting for socioeconomic status and study quality. The high heterogeneity in studies limits the ability to confirm the association.
Lönnroth et al. (2008) [43]	1961–2007	21 observational studies (HIC and LMIC)	Review the available evidence on the association between alcohol use and the risk of TB.	NA	Heavy alcohol consumption (≥40 g/day) or alcohol use disorder significantly increases the risk of active TB (RR 3.50, 95% CI: 2.01–5.93), with the risk slightly reduced (RR 2.94, 95% CI: 1.89–4.59) after excluding small studies. Subgroup analyses controlling for confounders did not significantly affect results or explain the heterogeneity across studies. This suggests that both alcohol-related social behaviours and its impact on the immune system contribute to the elevated TB risk in heavy drinkers.
Simou et al. (2018) [44]	2005–2018	49 studies (Asia, sub-Saharan Africa, America, and Europe)	Assess the association between alcohol consumption and risk of TB	Newcastle-Ottawa Scale scoring system, 24 studies were of high quality and 20 studies were of low-quality score.	Alcohol consumption increased TB risk by 1.90 (95% CI: 1.63–2.23) compared to no or low intake. Sensitivity analysis found a slightly lower risk (OR: 1.60, 95% CI: 1.39–1.84) with no alcohol as the reference. A pooled analysis of four studies reporting hazard ratios indicated a nearly three-fold increase in TB risk (HR: 2.81, 95% CI: 2.12–3.74). An exposure-response analysis showed a 12% increase in TB risk per 10–20 g of daily alcohol.

**Table 2:** Comparative synthesis of range of association, consistency across systematic review and potential programmatic implications of medical and behavioural determinants of Progression from TB Infection to TB Disease.

Risk Factor	Number of Reviews	Range of Association ^a^	Consistency Across Systematic Reviews ^b^	Potential Programmatic Implications
Diabetes mellitus	4	RR/OR: 1.5–3.59	Yes	Consider prioritised TBI screening, especially in poorly controlled DM
Malnutrition, iron and vitamin deficiency	1	HR: 2.23	NA	Integrate nutritional support with TB prevention
Vitamin D deficiency	1	aOR: 1.48–4.28	NA	Integrate Vitamin D support with TB prevention
Anaemia	1	HR: 137–3.56 (higher for severe anaemia)	NA	Consider anaemia control for people with TB
Cancers ^c^	2	IRR: 2.25–26	Yes	Targeted TBI screening in high-incidence settings
Chronic kidney disease (CKD)	2	HR: 1.57–11.35	Yes	Consider risk-based screening in advanced CKD
TNF-α inhibitors ^d^	6	RR/OR = 1.16–307.71	No	Pre-treatment TBI screening essential
Chronic obstructive pulmonary disease (COPD)	2	OR = 1.46–2.29	Yes	Individualised risk assessment
Smoking	4	RR: 1.46–3.41	Yes	Smoking cessation as TB prevention strategy
Alcohol use disorders	2	HR/RR: 1.90–3.50	Yes	Integrate alcohol reduction into TB control

^a^ Ranges reflect estimates reported in included systematic reviews and are not pooled effect sizes. HR = hazard ratio; IRR= incidence rate ratios; RR = relative risk; OR = odds ratio; ^b^ Consistency across systematic reviews (Yes, No or Not Applicable (NA, if only one review); ^c^ Cancers: Hematological cancers, head cancers, neck cancers, lung cancers, breast and other solid cancers, ^d^ TNF-α: Tumour necrosis factor; TNF-α inhibitors used to treat conditions like multiple sclerosis (MS), rheumatoid arthritis (RA), inflammatory bowel disease (IBD), and Systemic Lupus Erythematosus (SLE), impair granuloma formation.

## Data Availability

The data that support the findings of the study are available from one of the first authors (K.G.K.) upon reasonable request.

## Supplementary Materials

The following supporting information can be downloaded at: https://www.mdpi.com/article/10.3390/tropicalmed11040094/s1, Table S1: Preferred Reporting Items for Systematic reviews and Meta-Analyses extension for Scoping Reviews (PRISMA-ScR) Checklist—From Infection to Disease: The Role of Medical and Behavioural Determinants of Progression from TB Infection to TB Disease.
